# Attention-deficit hyperactivity disorder stigma: The silent barrier to care

**DOI:** 10.4102/sajpsychiatry.v28i0.1865

**Published:** 2022-12-08

**Authors:** Renata Schoeman, Tawni Voges

**Affiliations:** 1 Stellenbosch Business School, Stellenbosch University, Bellville, South Africa; 2 Private practice, Bellville, South Africa; 3 Goldilocks and The Bear Foundation, Bellville, South Africa; 4 Department of Psychology, Faculty of Social Sciences, Stellenbosch University, Cape Town, South Africa

**Keywords:** attention deficit hyperactivity disorder, stigma, barriers to care, children, well-being, discrimination, interventions, interviews

## Abstract

**Background:**

Attention-deficit hyperactivity disorder (ADHD) is the most common psychiatric disorder in childhood, with symptoms persisting into adulthood in 60% of individuals. If left untreated, the emotional, social and financial consequences can be dire, with many children and adults not reaching their full potential and having a reduced quality of life.

**Aim:**

The study explored parents’ and educators’ understanding and experience of stigma in relation to their children’s ADHD.

**Setting:**

Participants were recruited from six schools in the Cape Town metropole, in which the Goldilocks and The Bear Foundation (which delivers mental health services to underprivileged children) were active.

**Methods:**

A convergent parallel mixed methods research design (consisting of a quantitative survey and an in-depth interview component) was conducted to explore the lack of knowledge about ADHD and stigma as potential barriers to help-seeking behaviour, diagnosis and treatment for children with ADHD.

**Results:**

Instrumental barriers to care had a bigger impact on practical access to care, while attitudinal and stigma-related resources were found to have a significant impact on well-being of individuals. Core to the themes arising from the interviews were questions of how lack of knowledge influences stigma, how stigma materialises in discriminatory behaviour and how stigma acts as a barrier to care.

**Conclusion:**

The findings contribute to the literature by exploring parents’ and educators’ understanding and experience of stigma in relation to their children’s ADHD. A collaborative stakeholder approach is needed for effective, comprehensive and relevant interventions to combat stigma and enhance early identification of and interventions for ADHD.

**Contribution:**

In order to improve access to care, treatment, and well-being of individuals directly or indirectly affected by ADHD, it is crucial that stigma needs to be addressed.

## Introduction

Attention-deficit hyperactivity disorder (ADHD) is the most common psychiatric disorder in childhood, with a meta-analysis establishing the prevalence in school-aged children at 5.90%.^1^ Schoeman et al. reported a prevalence rate of 2.52% in a community sample of children in the Western Cape.^2^ Symptoms persist in up to 60% of adults, with 2.80% of adults meeting the criteria for ADHD.^3^ In the first South African study exploring the prevalence of adult ADHD, it was estimated that 1.09% of adults in the community and up to 52.5% of adults in clinical psychiatric settings have ADHD.^4^

Children and adults across all socio-economic, religious, cultural and racial groups are affected by ADHD, which can have far-reaching negative educational, social and emotional consequences.^5^ Limited access to diagnosis and treatment contributes to the direct, indirect and intangible cost of the disorder and has a negative impact on the quality of life of individuals with ADHD. As a result of the lack of child mental health services in South Africa,^6^ many children who potentially meet the diagnostic criteria for ADHD are not timeously identified and diagnosed, which in turn affects their opportunity to receive early intervention and reach their potential. The situation is further aggravated by stigma.

Stigma as a key barrier to be addressed to improve the quality and access to care in mental health has been highlighted in 2013 by the South African National Mental Health Policy Framework and Strategic Plan.^7^ Booysen et al. confirmed the negative impact of stigma, with participants in their Eastern Cape study describing stigma as a painful and distressing experience and a significant barrier to the inclusion of persons living with mental disorders in community activities, health care services, workplaces and educational institutions.^8^ In a community sample in KwaZulu-Natal, most parents attempting to access treatment for ADHD for their children had experienced stigma related to their children’s illness, and many parents also endorsed the existence of popular misperceptions about ADHD.^9^

There is a lack of knowledge regarding stigma as it relates to ADHD and how this in turn affects help-seeking behaviour. The study specifically explored parents’ and educators’ understanding and experience of stigma in relation to their children’s ADHD. The increased understanding of how stigma acts as a barrier to care in terms of diagnosis and treatment and its negative impact on well-being and quality of life could guide multistakeholder collaboration to identify effective culturally and contextually relevant initiatives to decrease stigma and enhance service provision.

## Literature review

The impact of ADHD on children and their families is significant – especially if left untreated. A recent meta-analysis confirmed the negative impact of ADHD on the quality of life of children and adolescents.^10^ While their physical functioning was only moderately impaired, emotional, social and school functioning were strongly impaired. A further meta-analysis confirmed that parents of children with ADHD experienced moderate impairment in their quality of life when compared with parents with neurotypical children.^11^ Studies have confirmed that individuals with ADHD are at increased risk of comorbid psychiatric disorders (including substance use disorders), accidental injuries, educational underachievement, unemployment, gambling, teenage pregnancy, difficulties socialising, delinquency, suicide and premature death.^12^ For both the children and their families, these difficulties associated with ADHD are exacerbated by stigmatisation.^13^

Stigma, since Goffman’s seminal work in 1963,^14^ is now widely recognised as a global phenomenon that poses a substantial threat to individual and societal functioning. Stigma operates at both macro and micro sociological levels by hindering individuals from accessing adequate health care, obstructing access to much-needed social support, exacerbating social anxiety and hampering societal integration.^15,16,17^ Stigma is a complex social phenomenon that involves processes of labelling, stereotyping, separation, status loss and discrimination.^18^

Pryor et al. propose a conceptual model of stigma.^19^ They refer to public stigma as being the consensual understanding that a social attribute is devalued, as manifested in cognitive, affective and behavioural reactions to someone they perceive to have a stigmatised condition. Self-stigma is the apprehension of being exposed to stigmatisation and the potential internalisation of the negative beliefs and feelings associated with the stigmatised condition. Stigma by association reflects social and psychological reactions to family and friends associated with a stigmatised person, as well as people’s reactions to being associated with a stigmatised person. Finally, structural stigma is the legitimatisation and perpetuation of a stigmatised status by society’s institutions and ideological systems. Stigma thus seems to act as a barrier to care.

Reported barriers to adequate mental health care in South Africa include socio-economic hardship, lack of knowledge and insight, lack of family support, embedded cultural beliefs about mental health, stigmatisation, lack of health care resources and infrastructure and poor access to technology and information.^20,21^ A lack of understanding of the causes of mental health disorders, such as ADHD, can cause a child’s behavioural difficulties to be attributed to acting up or to believing that a child is behaving in a certain way owing to insufficient parenting and discipline.^22^ The way in which individuals conceptualise and understand mental health conditions thus influences the manner in which they access care. Also, certain cultural and belief systems may attribute psychiatric symptoms to malevolent interventions such as bewitchment, sorcery and magic and the influence of departed ancestors who wish to correct inappropriate social behaviour, as described by Atindanbila et al.^23^ In exploring community perceptions in the Limpopo province, Sehoana^24^ found that the Pedi community’s perception of mental illness is influenced by cultural and religious beliefs, which in turn affect the choice of treatment, such as seeking traditional or spiritual healing rather than mainstream Western psychiatric treatment.

However, stigma is not only a community response. Studies conducted in mental health care settings have reported the prevalence of discrimination experienced by mental health care users ranging from 16% to 44%.^25^ Furthermore, Fox et al.^26^ reported that self-stigma appears to be the most incapacitating part of stigma. Self-stigma negatively affects self-efficacy, self-esteem and hope and is associated with decreased empowerment, weakened social support and diminished quality of life. The likelihood of these stigmatised individuals seeking mental health care, as well as the likelihood of adhering to pharmacotherapy, therefore decreases. Oexle et al.^27^ found that being diagnosed and labelled with a mental health diagnosis carried a higher risk of suicidal ideation, owing to perceived stigma increasing secrecy and hopelessness. It is therefore crucial to understand stigma and the impact thereof on the diagnosis and management of ADHD.

Addressing stigma has also been identified as a key element of the World Health Organization’s^28^ goal:

‘[*A*] world in which mental health is valued, promoted and protected, mental disorders are prevented, and persons affected by these disorders are able to exercise the full range of human rights and to access high quality, culturally-appropriate health and social care in a timely way to promote recovery, in order to attain the highest possible level of health and participate fully in society and at work, free from stigmatization and discrimination.’ (p. 9)

## Methods

A convergent parallel mixed methods research design (consisting of a quantitative survey and an in-depth interview component) was conducted to explore the lack of knowledge about ADHD and stigma as potential barriers to help-seeking behaviour, diagnosis and treatment, for children with ADHD. Yardley et al. suggested a pragmatic worldview as a guide in selecting a mixed methods study.^29^ Pragmatism proposes that qualitative and quantitative methods complement each other, where quantitative enquiry provides norm-referenced data that can be compared with different people and populations, while qualitative enquiry provides rich information about individual experiences. In this article, we are reporting on the qualitative findings of the study. Quantitative findings are discussed in a separate article.

### Study population and sampling strategy

Participants were recruited from six schools in which the Goldilocks and The Bear Foundation is active in delivering mental health services to underprivileged children with the aim of removing mental health barriers to education. In these schools, 451 children were screened by the organisation for the presence of emotional, behavioural or learning disorders. The parents of these 451 children were invited to participate in the study. Furthermore, the 163 educators at these schools were also invited to take part.

A total of 26 parents and 19 educators completed the questionnaire about the barriers to access to care. This represents a response rate of 5.76% of parents and 11.66% of teachers. Of these, 12 parents (2.60% of the invitees, 46.00% of the participants) and six educators (3.68% of the invitees, 32.00% of the participants) agreed to take part in a telephonic interview to gain a deeper understanding of their experiences regarding ADHD and stigma in their community.

### Data collection

All participants completed the Barriers to Access to Care Evaluation (BACE-3) originally designed by Clement et al.^30^ The BACE-3 is a 30-item self-administered questionnaire and explores stigma-related barriers such as anticipated discrimination, social stigma, disclosure concerns, stereotypes, internalised stigma and stigma by association. The authors demonstrated that the measure has acceptable test–retest reliability, validity, acceptability and readability, with an average 11 or 12-year-old being able to understand the questionnaire.^30^

Using a Portuguese translation of the BACE-3, Miranda et al. reported good validity, with an internal consistency of 0.961 for the full BACE-3 scale and 0.964 for the stigma subscale.^31^

Semistructured telephonic interviews were conducted between September and October 2020. Three open-ended questions were posed to participants, with probes that were needed to extend the answers provided without leading the desired response. Questions were related to parents’ and educators’ understanding of stigma and their experience of stigma in relation to ADHD, and these aimed at gaining a deeper understanding of how stigma acts as a potential barrier to accessing care. All interviews were recorded once respondents had given permission for the recording, as specified in the ethical procedures for this study.

### Data analysis

Descriptive statistics and correlations were used to explore the participants’ demographics and the barriers that they experienced in accessing care based on the BACE-3. The detailed results are reported in a separate article. Recorded interviews were transcribed by the researcher and checked for accuracy by an independent researcher (who was also bound to confidentiality), followed by a qualitative analysis of the data by means of ATLAS.ti (version 8), a computer-aided qualitative data analysis software (CAQDAS) programme.^32^ Braun et al.’s approach to reflexive thematic analysis was used, which provides a rigorous and systematic approach to analysing qualitative data by uniting seemingly disparate pieces of data into meaningful patterns to generate codes and themes.^33^ The first phase was to read through the data to become familiar with the content and to obtain an overview. Thereafter, dual descriptive-level (level 1 open-coding, where segments of meaning were identified, and level 2 free-coding) and conceptual-level analysis (where related codes were categorised into groups and where relationships between categories were sought to identify and name themes) followed. The final phase consisted of revisiting the research questions, themes, subthemes and notes to connect existing research and literature on the topic of interest and incorporating this into the written results and discussion.

By interview 13, data saturation had been sensed. To protect against researcher bias, the independent researcher randomly checked the primary researcher’s coding and theme formation. A meeting was held to audit the process. The feedback was used to ensure the quality of the set of interviews and analysis thereof.

### Ethical considerations

The project was approved by the university’s Research Ethics Committee (ref. no. REC-2020-13255) and the provincial Department of Education (ref. no. 20200220-4736). All participants provided voluntary informed consent for participation.

## Results and discussion

### Barriers to access to care

Forty-five participants completed the BACE-3. Participants were on average 40.6 years old (s.d. = 10.50 years), with ages ranging from 22 to 64 years. One of the participants had no formal education, nine (25%) had completed matric and 25 (55.6%) had some or had completed their tertiary education. Twelve (26.6%) participants were unemployed.

Ten (22%) participants reported that they had been diagnosed with a mental health condition, of which seven indicated that they had been diagnosed with a mood disorder. Seventeen (38%) of the participants reported that their child had been diagnosed with a mental health condition – of which 14 were diagnosed with ADHD, with or without comorbid psychiatric disorders.

The BACE-3 clusters barriers to care into three groups. Instrumental barriers refer to practical and logistical factors that prevent people from accessing care (e.g. a lack of financial means or transport). Attitudinal barriers refer to misconceptions that discourage individuals from seeking help, such as the belief that medication is dangerous. Stigma-related barriers refer to perceived public stigma and the resultant self-stigma (e.g. concerns about others’ opinions on the individual receiving a diagnosis or seeking treatment).

The most frequently reported barrier was not being able to afford the financial costs involved in accessing treatment (mean = 2.4, s.d. = 1.19), which represents an instrumental barrier to care. This was followed by participants wanting to solve the problems themselves (mean = 2.3, s.d. = 1.25) and concerns about the treatments available (mean = 2.1, s.d. = 1.16), which both represent attitudinal barriers. Concern that the participant or their child may be seen as weak for having a mental health problem was the most frequently reported stigma-related barrier (mean = 2.1, s.d. = 1.04). A least significant difference post hoc test indicated a significant difference between the instrumental and stigma subscales (*p* = 0.05). The mean scores for the instrumental and stigma subscales were 1.70 (s.d. = 0.66) and 1.55 (s.d. = 0.67), respectively.

### Understanding stigma

Eighteen telephonic interviews were conducted. Fifteen participants (83%) were women, and 10 (56%) participants were parents (or educator-parents) to a child with ADHD.

The final code list consisted of 32 codes, representing 296 comments. The codes were then grouped into 10 subthemes, from which four key themes emerged: understanding stigma, factors influencing access to care, unmet needs and desires in the community and suggestions for interventions. Core to these four themes is stigma: how lack of knowledge influences stigma, how stigma materialises in discriminatory behaviour and how stigma acts as a barrier to care.

Although the BACE-3 responses indicated that instrumental barriers to care had a bigger impact on the practical access to care, attitudinal and stigma-related resources were found to have a significant impact on the well-being of individuals.

#### Stigma defined

In making sense of stigma, the interviewees outlined three processes that take place. Firstly, a difference is recognised in a person. Then, based on this difference, a label is applied to the person. Finally, once this label has been attached, the person loses their uniqueness, being represented instead by the label.

Instances of stigma rest firstly on the premise of recognition of what Goffman refers to as an ‘undesired difference’ in a person, which results in such a person experiencing feelings of shame and lack of acceptance.^14^ Many of the study interviewees described experiences of difference related to stigma, either in their child or in society in general.

‘… somebody’s either labelled or treated differently because there’s something that people don’t understand about them. It could be something physical, it could be something behavioural, but it’s just there will be a difference somehow.’ (Participant 12, Parent, Male, Comment 9)

Secondly, by applying a label to a person, a connection is made between that person and one or more stereotypes,^18^ which then links the person to a stereotype.^34^

‘… that is like it’s a label that they use, like usually stereotyping about what ADHD is and, you know, saying that they’re dumber – using that word, that they dumb. Or their parents don’t give them enough attention; that is why they are that way.’ (Participant 1, Parent, Female, Comment 13)

A third theme that emerged during the interviews was that the process of stigmatisation leads to a loss of identity or individuality.

‘It means that, to me, that generally people put a name or categorises someone in a group, instead of looking at people individually. So they will judge according to a sickness or disorder or whatever, instead of looking at people individually.’ (Participant 15, Educator, Female, Comment 9)

#### Manifestations of stigma

Conceptualisations of stigma remained broad and encompass many different experiences and potential consequences. While experiences of the interviewees were varied, common themes emerged, including:

- bullying (Participant 2, Parent, Female, comment 13 and Participant 3, Parent, Female, Comment 11)
- exclusion (Participant 1, Parent, Female, Comment 7 and Participant 3, Parent, Female Comment 19)
- being overlooked and misunderstood (Participant 1, Parent, Female, Comment 16; Participant 3, Parent, Female, Comment 13 and Participant 16, Educator, Female, Comment 1)
- individual needs not being addressed (Participant 1, Parent, Female, Comment 45; Participant 2, Parent, Female, Comment 4; Participant 4, Parent, Female, Comment 34; Participant 11, Parent, Male Comment 4; Participant 15, Educator, Female, Comment 5 and Participant 17, Educator, Female, Comment 16) and
- having behaviour interpreted through a lens (Participant 4, Parent, Female, Comment 17; Participant 8, Parent, Female, Comment 28 and Participant 9, Parent, Female, Comment 4).

This finding confirms previous research regarding the vulnerability of children with ADHD to being bullied, as well as the propensity of individuals with mental health disorders to be excluded from social activities, from opportunities for self-development, and in decision-making regarding their treatment.^35,36,37^

An interesting tension was observed where some parents felt that their children should be treated differently in order to accommodate their special needs, whereas other parents felt their children’s emotional needs would be better accommodated by being treated in the same way as other children in their class.

#### Impact of stigma on the individual

Stigma affects individuals with mental health disorders emotionally, psychologically and materially. Findings of this study indicated that stigma led to:

- decreased self-esteem and self-worth (Participant 1, Parent, Female, Comment 18 and Participant 11, Parent, Male, Comment 34)
- increased isolation (Participant 5, Parent, Female, Comment 13 and Participant 11, Parent, Male, Comment 16) and
- acting-out behaviour such as truancy and aggression (Participant 7, Parent, Female, Comment 34).

This interaction between social isolation and stigma seems to be bidirectional inasmuch as experiences of stigma may lead people to isolate others who are different, and conversely, stigmatised people may isolate themselves for fear of being discriminated against.

#### Impact of stigma on the family

Findings of this study confirmed the literature that indicates that parents of children with ADHD are at an increased risk of depression, anxiety and social isolation. These disorders have been attributed to challenging behaviour of the child, which is compounded by stigma.^38^

Courtesy stigma (when an individual is mistreated because of their relationship with a stigmatised person) and affiliate stigma (when individuals perceive and internalise courtesy stigma) clearly affected families in this study. These stigmas have an impact on:

- families’ day-to-day living (Participant 5, Parent, Female, Comment 11; Participant 5, Parent, Female, Comment 14; Participant 9, Parent, Female, Comment 9 and Participant 10, Parent, Male, Comment 5)
- they also contribute to emotional hardship for parents (Participant 1, Parent, Female, Comment 20; Participant 4, Parent, Female Comment 22 and Participant 15, Educator, Female, Comment 11).

‘We generally don’t go to shops if it’s busy; we generally try to avoid busy areas, or busy shopping centres or certain – even going to religious establishments, for example, because you need to be quiet; you need to meet certain requirements to be able to sit through a service. You understand what I’m saying? And that is why we tend to just rather stay at our home and rather go at peculiar times to the shop so that we can avoid people looking at us in a certain way.’ (Participant 10, Parent, Male, Comment 5)

‘You’re exhausted of the day and then you don’t want to show them you’re crying, because then you look so weak … but when I get into bed, it’s like, you know, I just feel for crying, I just feel like … sometimes I’m washing up and then my tears just roll down, because there’s no one to help you; there is no one to support you; there is no one.’ (Participant 4, Parent, Female, Comment 22)

This finding confirmed the contention of Bussing et al. that, while the entire family may be affected by courtesy stigma, it is usually the mothers who carry the main responsibility of caring for the child with ADHD and are thus more strongly affected by the stigma, as well as ‘mother-blame’.^39^ This was echoed by one participant:

‘It will affect the family. If you look at it in that way, it will affect the family. Maybe not the father, but the mother. The mother will feel bad. It will, like I said now, it will affect the child and it will affect the parents also. For the stigmas that is in the community, because of the name-calling and stuff like that, maybe not the father so much. You see? But in rural communities, it will affect the mother. She’ll feel bad for her child also.’ (Participant 5, Parent, Female, Comment 14)

### Addressing stigma

In investigating psychiatric stigma, Egbe^40^ describes that discriminatory behaviour and negative attitudes are based on society’s perception of a mental healthcare user as diverging from the group. *Normalising mental health conditions thus could potentially lead to a decrease in stigma*, as confirmed by the interviewees (Participant 8, Parent, Female, Comment 31; Participant 14, Educator, Female, Comment 20 and Participant 18, Educator, Female, Coment 11).

‘What they should know is that the people with mental disability or mental health issues are just as normal and are also just as much human as the person that is perceived as normal.’ (Participant 8, Parent, Female, Comment 31)

*For normalising to occur, awareness and knowledge about ADHD need to be increased* (Participant 1, Parent, Female, Comment 28; Participant 3, Parent, Female, Comment 16; Participant 2, Parent, Female, Comment 20; Participant 8, Parent. Female, Comment 21, Participant 10, Parent, Male, Comment 8; Participant 11, Parent, Male, Comment 13; Participant 12, Parent, Male, Comment 6 and 14; Participant 13, Educator, Female, Comment 11 and Participant 14, Educator. Female, Comment 15).

‘People don’t know. They are not informed. They don’t know. They are not aware. So people would have treated people differently if there was more information available for them … They don’t understand. They don’t understand. And it will take quite some time for people to get to that stage.’ (Participant 11, Parent, Male, Comment 13)

The normalising of mental health conditions was seen by interviewees as a *responsibility to be shared* by parents, educators and government, although the role of schools and educators herein was specifically accentuated (Participant 3, Parent, Female, Comment 16; Participant 14, Educator, Female, comment 19 and Participant 15, Educator, Female, Comment 18).

Interviewees also expressed a wish for *enhanced societal empathy* (Participant 1, Parent, Female, Comment 34; Participant 2, Parent, Female Comment 34 and Participant 9, Parent, Female, Comment 18), which aligns with Hinshaw et al. who consider empathy enhancement as a key component of successful and long-lasting stigma reduction strategies.^41^

‘Don’t treat the children differently and treat them like normal kids, and so on. Just be softer on them and gentle on them … You mustn’t just be breaking them down and stuff; you must pull him up.’ (Participant 2, Parent, Female, Comment 21)

‘People just needs to be a little sensitive in talking or dealing with a specific person. Sometimes people will say and do things that is really hurtful.’ (Participant 9, Parent, Female, Comment 18)

## Conclusion

Attention-deficit hyperactivity disorder is a recognised and common mental health disorder with far-reaching implications for individuals and their families. However, the disorder often acts as a barrier to accessing care, with a negative impact on whole families’ well-being and quality of life. In this study, interviewees described their understanding of stigma in relation to ADHD as a three-phase process: recognising a difference in a person, labelling that person and consequent deindividuation of that person. According to data collected in this study, the process resulted in different manifestations of public stigma (which result in exclusion), self-stigma (which leads to poor self-esteem) and stigma by association (which culminates in emotional hardship and isolation).

Drawing on their experience with ADHD stigmatisation, the interviewees expressed a need for intervention in the community on multiple levels to enhance the lives of children and families living with ADHD. Their responses made it clear that any intervention needs to be multifaceted (e.g. addressing the different barriers to care, improving knowledge about ADHD and enhancing empathy) and multilevel (addressing public stigma, self-stigma and stigma by association).

Previous studies confirmed that in the absence of fundamental change in addressing both the deeply held attitudes and beliefs about ADHD and in changing the contextual circumstances, interventions targeted at only one mechanism at a time would ultimately fail.^42^ The effectiveness of narrow, targeted interventions (e.g. a media campaign) would be undermined by contextual factors (e.g. the educational and health systems) if these were not also addressed.

A limitation of this study was the relatively low level of participation. It has to be noted that the study was conducted during the coronavirus disease 2019 (COVID-19) pandemic and lockdown. The study was initially planned to consist of in-person interviews and focus group discussions, which, in this specific population, would have led to better engagement. However, the investigators had to resort to telephonic interviews according to the Department of Education’s guidelines for conducting research during the lockdown period.

Few studies across the world have focused on either the service user’s perspective on stigma and discrimination or on the behaviour domain of behavioural change by people with or without mental illness in the complex processes of stigmatisation. This study contributes to the literature by exploring parents’ and educators’ understanding and experience of stigma in relation to their children’s ADHD. The study also stresses the need for effective, comprehensive and relevant interventions, not only in the community but also in the educational and health environments, to decrease stigma and enhance early identification of and interventions for ADHD.
